# Imaging bacteria with radiolabelled quinolones, cephalosporins and siderophores for imaging infection: a systematic review

**DOI:** 10.1007/s40336-016-0185-8

**Published:** 2016-07-18

**Authors:** S. Auletta, F. Galli, C. Lauri, D. Martinelli, I. Santino, Alberto Signore

**Affiliations:** 1 Nuclear Medicine Unit, Department of Medical-Surgical Sciences and of Translational Medicine, Faculty of Medicine and Psychology, St. Andrea Hospital, “Sapienza” University of Rome, Via di Grottarossa 1035, 00189 Rome, Italy; 2 Microbiology Unit, Department of Clinical and Molecular Medicine, “Sapienza” University of Rome, Rome, Italy

**Keywords:** Antibiotics, Infection, Bacteria, Radiolabelled antibiotic, Molecular imaging

## Abstract

Bacterial infections are still one of the main causes of patient morbidity and mortality worldwide. Nowadays, many imaging techniques, like computed tomography or magnetic resonance imaging, are used to identify inflammatory processes, but, although they recognize anatomical modifications, they cannot easily distinguish bacterial infective foci from non bacterial infections. In nuclear medicine, many efforts have been made to develop specific radiopharmaceuticals to discriminate infection from sterile inflammation. Several compounds (antimicrobial peptides, leukocytes, cytokines, antibiotics…) have been radiolabelled and tested in vitro and in vivo, but none proved to be highly specific for bacteria. Indeed factors, including the number and strain of bacteria, the infection site, and the host condition may affect the specificity of tested radiopharmaceuticals. Ciprofloxacin has been proposed and intensively studied because of its easy radiolabelling method, broad spectrum, and low cost, but at the same time it presents some problems such as low stability or the risk of antibiotic resistance. Therefore, in the present review studies with ciprofloxacin and other radiolabelled antibiotics as possible substitutes of ciprofloxacin are reported. Among them we can distinguish different classes, such as cephalosporins, fluoroquinolones, inhibitors of nucleic acid synthesis, inhibitors of bacterial cell wall synthesis and inhibitors of protein synthesis; then also others, like siderophores or maltodextrin-based probes, have been discussed as bacterial infection imaging agents. A systematic analysis was performed to report the main characteristics and differences of each antibiotic to provide an overview about the state of the art of imaging infection with radiolabelled antibiotics.

## Introduction

Bacterial infections are still one of the main causes of mortality and morbidity worldwide. This is also because of the lack of specific agents to detect infective foci or to discriminate infection from sterile inflammation. Diagnostic radiological imaging offers various techniques to identify inflammatory processes, but they allow to detect only anatomical changes of the infection and are not always able to discriminate infections from normal postsurgical changes in the early stages [1]. On the other hand, nuclear medicine offers many radiopharmaceuticals that can detect physiological and biochemical changes at the early stages of infection. They include radiolabelled antimicrobial peptides, antibiotics, leukocytes, but also immunoglobulins and cytokines labelled with gamma- or positron-emitting isotopes (^18^F, ^99m^Tc, ^111^In, ^67^Ga etc.…) [2–5]. In addition, the use of radiopharmaceuticals able to detect T lymphocyte infiltration in autoimmune or inflammatory bowel diseases (IBD) has been proposed as an alternative approach [6]. Unfortunately none of these are specific enough for bacteria thus allowing to discriminate infection from sterile inflammation, in spite of high sensibility. This depends on the nature of the radiopharmaceutical, its biodistribution and binding properties but also on the type of microorganism, the kind of infection, the infection site and the host conditions. Another unsolved issue is the minimum number of micro-organisms necessary to perform a reliable diagnosis, which has already been discussed [7]. In clinical nuclear medicine, among the many ^99m^Tc-labelled compounds, antibiotics looked the most promising to image infection. They are divided in several classes, based on their mechanism of action. The first radiolabelled antibiotic, used as radiopharmaceutical, was ^99m^Tc-ciprofloxacin, that pioneered the use of radiopharmaceuticals for bacterial imaging. Nevertheless, it appeared soon clear that the task of imaging bacteria is very complex with many problems to be solved [7–9].

In this article, the use of radiolabelled ciprofloxacin is reviewed together with other “infection-specific” radiolabelled antibiotics, developed with the aim to discover tools with better properties than ^99m^Tc-ciprofloxacin. These antibiotics are divided into several categories, according to their mechanisms of action.

## Bacteria, biofilm and antibiotic mechanisms of action

Planktonic bacteria are free-living bacteria, which are generally treatable with antibiotics but when they adhere to a surface develop a biofilm. A commonly used definition of a biofilm is a “microbially derived sessile community characterized by cells that are irreversibly attached to a substratum, interface or to each other, are embedded in a matrix of extracellular polymeric substances that they have produced, and exhibit an altered phenotype with respect to growth rate and gene transcription” [10]. Biofilm embedded bacteria represent a serious clinical problem in medicine, because their infections are notoriously difficult to treat due to extreme resistance to antibiotics.

Antibiotics are drugs of natural or synthetic origin that have the capacity to kill (bactericidal drugs) or inhibit (bacteriostatic drugs) the cell growth. Most bactericidal antimicrobials are: cephalosporins, carbapenems, glycopeptides, fluoroquinolones, polymyxins that inhibit DNA synthesis, RNA synthesis, cell wall synthesis, or bacterial protein synthesis.

Fluoroquinolones (FQs) are bactericidal antibiotics effective for both Gram-negative and Gram-positive bacteria and ciprofloxacin is the most widely used antimicrobial agent among FQs. The action of ciprofloxacin results from inhibition of the enzymes topoisomerase II (DNA gyrase, gyrA and B) and topoisomerase IV (grlA and B), which are required for bacterial DNA replication, transcription, repair, strand super coiling repair, and recombination. Resistance to FQs in bacteria is mainly mediated by alterations in DNA gyrase and topoisomerase IV with specific amino acid substitutions in the “quinolone-resistance determining region” (QRDR) in gyrA and B subunits of DNA gyrase and parC and parE subunits of topoisomerase IV. Other common mechanisms are reduced permeability/increased efflux of ciprofloxacin across bacterial membranes, and plasmids that protect cells from the lethal effects of FQs [11, 12, 15].

Toxic effects of FQs on humans have been attributed to their interactions with different receptor complexes, such as blockade of the GABAa receptor complex within the central nervous system, leading to excitotoxic type effects and oxidative stress.

The cephalosporins are the largest family of β-lactam antibiotics. They are bactericidal agents and have the same mode of action as other beta-lactam antibiotics (such as penicillin). Cephalosporins disrupt the synthesis of the peptidoglycan layer of bacterial cell walls by binding to penicillin binding proteins (PBPs), causing the walls to break down and eventually the bacteria die. The three fundamental mechanisms of antimicrobial resistance are: enzymatic degradation of antibacterial drugs, changes in PBPs, and changes in membrane permeability to antibiotics. The most important mechanism of resistance to cephalosporins is destruction of beta-lactam rings by β-lactamase enzymes. Mutational changes in original PBPs or acquisition of different PBPs will lead to inability of the antibiotic to bind to the PBPs and inhibit cell wall synthesis. A change in the number or function of the general diffusion porin channels can reduce the permeability.

Since antimicrobial compounds act on processes that are unique to bacteria, it has been proposed that radiolabelled antibiotic should be able to distinguish microbial from non microbial inflammation, because of their specific binding to the causative agents.

## Ciprofloxacin

^99m^Tc-ciprofloxacin, also known as Infecton, was the first radiolabelled antibiotic tested in human to image infections [8]. In preclinical studies many different animal models have been used to prove ciprofloxacin specificity. In rats ^99m^Tc-ciprofloxacin showed an excellent biodistribution with renal clearance, and targeting experiments showed a high sensitivity but low specificity. Ciprofloxacin was also conjugated with propylamine and then labelled with ^68^Ga, revealing to be a good bacteria-specific imaging agent in a *S. aureus* infected rat model [16–18].

Different results were obtained when ^99m^Tc-ciprofloxacin was studied in mice and both high sensitivity and specificity for imaging infections were obtained [14, 19–22].

Controversial results were obtained using other animal models like rabbits, camelids, dogs or swines to evaluate the ability of ^99m^Tc-ciprofloxacin to localize the infectious site, in severe acute pancreatitis, prosthetic joint infections or other suspected infections [23–26].

In clinical studies, it was more difficult to study the pharmacokinetics of ciprofloxacin in organs and tissues, particularly in the gastrointestinal tract, lungs and soft tissues.

Positron Emission Tomography (PET) could be a technique that allows a direct quantification of the antibiotic, when labelled with positron-emitting isotopes like ^18^F. Indeed, two studies performed by Brunner et al. and Langer et al. [13, 27], using PET with ^18^F-ciprofloxacin, showed opposite results in healthy volunteers and patients with suspected infections, respectively. In particular Langer and colleagues concluded that ^18^F-ciprofloxacin is not a suitable and specific radiopharmaceutical for imaging infections.

Many other studies in patients have been performed using ^99m^Tc-labelled ciprofloxacin. Most of them had concordant results about the labelling procedure using the kit formulated at St. Bartholomew’s Hospital in London [28] and about the metabolism of the radiopharmaceutical, which was prevalently renal, with low level of hepatic uptake and no bone marrow, bone and gastrointestinal uptake. However, final results showed a high variability in terms of sensibility and specificity. These controversial and variable data may depend on the type and site of infections, strain of micro-organisms, presence of antibiotic therapy, lack of standardized imaging parameters and interpretation criteria, but also on the type of imaging modality (SPECT or planar scintigraphy) [29, 30]. Some authors have considered Infecton as a good bacterial infection imaging agent, particularly when SPECT images are acquired for the diagnosis of pulmonary or extrapulmonary tuberculosis, fever or unknown origin (FUO), osteomyelitis, hip or knee prosthesis, active spinal infections, abdominal or gastrointestinal and orthopaedic infections, despite of conflicting results based on the type of infection. Moreover, it allows to evaluate the presence of infection in immune-suppressed patients, when white blood cell (WBC) imaging was uncertain or to monitor and optimize the antimicrobial treatment. However, in addition to image analysis, a microbiological culture was often useful to confirm the presence and nature of the infection [8, 28, 31–44]. Other authors have considered ^99m^Tc-ciprofloxacin as a potential imaging agent only for the diagnosis of orthopaedic infections, vertebral infections, osteoarticular tuberculosis and diabetic foot infections, in comparison to ^99m^Tc-WBC or immunoscintigraphy, showing excellent diagnostic accuracy [45–50]. By contrast, other studies, by Dumarey et al., De Winter et al., Sarda et al., Pucar et al., Appelboom et al. and Gemmel et al. [51–56], reported a low specificity but high sensitivity for Infecton imaging. These studies were performed in patients with different kind of infections and images were acquired and analyzed with different methods, but all concluded that ^99m^Tc-ciprofloxacin is unable to discriminate bacterial infection from sterile inflammation.

Finally, Zhang et al. [57, 58] performed a study with ciprofloxacin dithiocarbamate labelled with [^99m^TcN]^2+^ intermediate or [^99m^Tc(CO)_3_(H2O)_3_]^+^ intermediate. These radiopharmaceuticals were tested in *S. aureus* infected mice to evaluate their biodistribution and their ability to distinguish septic and aseptic inflammation in comparison to ^99m^Tc-ciprofloxacin. Experimental data showed that both new radiopharmaceuticals had a better target-to-non target (*T*/NT) ratio than ^99m^Tc-ciprofloxacin and they could be considered potential infection imaging agents.

## Fluoroquinolones

The quinolones can be differentiated in several generations, which differ for broad-spectrum activity and pharmacokinetic properties like a rapid and complete absorption from gastrointestinal tract or oral administration [59, 60].

For example pefloxacin is a fluoroquinolone antibacterial agent, which has been investigated as a potential substitute for ciprofloxacin in the detection of bacterial infections. It was labelled with ^99m^Tc, tested in mice infected with *E. coli* or injected with turpentine oil as sterile inflammation. Experimental data showed a main excretion through liver and intestine and a high retention in infectious foci than aseptic foci after 24 h from injection because of its specific binding to gyrase, confirmed by the *T*/NT ratio equal to 5.6 at 24 h post injection. Moreover pefloxacin had a rapid clearance, no accumulation in non-target organs, no toxicity, low cost and a simple preparation, that makes it a good potential imaging agent [61].

The second generation of fluoroquinolones includes many compounds, more or less specific for bacterial infections. Amongst the most specific agents there are lomefloxacin and ofloxacin that were always studied in comparison to ciprofloxacin. The radiolabelling procedure with ^99m^Tc is easy, without any purification in comparison to ciprofloxacin and they have been tested in *S. aureus* infected rats compared to normal rats as control. The biodistribution studies, obtained by ex vivo γ-counting, revealed renal excretion and low uptake in the liver, that indicates few hydrolyzed products of ^99m^Tc for both antibiotics.* T*/NT ratio for lomefloxacin was higher than for ofloxacin, 6.5 ± 0.5 and 4.3 ± 0.6 respectively, suggesting that lomefloxacin might be a better imaging agent than ofloxacin [62]. The low specificity of ofloxacin has been confirmed in another study performed by Erfani et al. They labelled the antibiotic with ^99m^Tc and investigated the biodistribution in *S. aureus* infected mice. Also in this case authors found a renal and liver clearance and a* T*/NT ratio equal to 2.02 ± 0.12 at 4 h after injection, a sign of poor specificity [63].

Two other poorly specific antibiotics are enrofloxacin and norfloxacin. The former was studied in comparison to ciprofloxacin by Siaens et al. It was radiolabelled with ^99m^Tc and injected in *S. aureus* treated rats. In this study the control rats were injected with turpentine oil, heat killed *S. aureus* or *C. albicans*. Results showed high renal uptake and no significant differences in the level of accumulation in the various inflamed muscles, indicating poor capacity to recognize infection from sterile inflammation [64]. Recently, ^99m^Tc-enrofloxacin was also studied by Shahzad et al. [65], obtaining more or less the same results as previously published by others. Indeed, the radiolabelled compound always showed the same biodistribution in non target organs and no high uptake in the infected muscle versus control.

The other non specific antibiotic, norfloxacin, was also labelled with ^99m^Tc and its biodistribution evaluated in rats infected with 10^7^–10^8^ CFU of *S. aureus*, heat killed *S. aureus* and turpentine oil. ^99m^Tc-norfloxacin has an excretion through the urinary system and the uptake in infected or non-infected muscles is not statistically different. Based on these data, it was concluded that norfloxacin cannot discriminate bacterial infection from sterile inflammation [66]. However, controversial results about norfloxacin were recently reported by Sazonova et al. [67] in rats where infection was induced with 10^9^ CFU of *S. aureus*. Turpentine oil was used as control. Their results showed a mild uptake in the infected muscle as compared to inflamed one. The* T*/NT ratios were 2.87 ± 0.80 and 1 ± 0.14, respectively, for infected and inflamed muscle, confirming that this radiopharmaceutical requires further studies to improve its specificity. Another study, performed by Zhang et al. [68], tested norfloxacin dithiocarbamate as a potential imaging agent. It was labelled with ^99m^Tc and the biodistribution was studied in *S. aureus* infected mice, while sterile inflammation was induced using turpentine oil. Experimental data revealed a main hepato-biliary clearance and the* T*/NT ratios were 3.46 and 1.23 at 3 h post-injection, respectively for bacterial infection and sterile inflammation.

With third-generation FQs several properties were improved through modifications of the quinolone nucleus, such as anti-microbial activity and pharmacokinetics [69].

An antibiotic of this category, that could be a substitute of ciprofloxacin, is sparfloxacin. It was labelled with ^99m^Tc and then biodistribution was studied in rats where infection was induced using 10^5^-10^6^ CFU of *S. aureus*. Biodistribution studies showed a rapid clearance through the urinary system and a high accumulation in the infection site, more than ciprofloxacin. As early as 2 h post-injection, the *T*/NT ratio was 5.10 ± 0.4 for sparfloxacin and 3.60 ± 0.4 for ciprofloxacin [9]. It is also remarkable that in this study very few CFU of *S. aureus* were used (only 10^5^–10^6^) as compared to the majority of published studies ranging from 10^7^ to 10^10^ CFU.

Levofloxacin is another third-generation fluoroquinolone. Shahzad et al. [70] labelled this antibiotic with ^99m^Tc using a freeze-dried kit. Biodistribution was studied in rabbit, infected with two different strains of bacteria (3 × 10^8^ CFU of *E. coli* and *P. aeruginosa*). Results showed kidneys as the main excretion route and* T*/NT ratios were 8.09 and 1.3 at 1 h post-injection, respectively in *P. aeruginosa* and *E. coli* infected muscles showing high variability depending on the kind of bacteria. Therefore ^99m^Tc-levofloxacin could be a promising imaging agent for lung, sinus bone and skin infections, but it also needs other studies.

A fluoroquinolone derivative that is able to distinguish between septic and aseptic inflammation is rufloxacin. It was always labelled with ^99m^Tc and the biological distribution was evaluated in Albino mice after induction of infection with live *E. coli* and inflammation with turpentine oil or heat killed *E. coli*. Experimental data revealed an excretion through kidneys and urine and the uptake in the infected muscles were higher than heat-killed bacteria and turpentine oil inflamed muscle. The* T*/NT ratio was also higher compared to ciprofloxacin at all time points (8.5 ± 0.1 vs 3.6 ± 0.4 3 h post injection), demonstrating that rufloxacin could be a good infection imaging agent [71].

Another third generation fluoroquinolone is fleroxacin that it was studied as a PET radiopharmaceutical by Fischman et al. [72]. It was labelled with ^18^F and its pharmacokinetics was evaluated in healthy and *E. coli* infected rabbits, mice and rats. Biodistribution showed a main excretion through the intestinal tract, then liver and kidneys and no accumulation in the brain, especially in rats and mice. Unfortunately the accumulation in healthy and infected muscle of all animals was similar and ^18^F-fleroxacin was considered a poor PET imaging agent for bacteria.

Compared to previous generation, the fourth generation of FQs has the advantage to be resistant to spontaneous mutation, reducing the risk of antibiotic resistance. Their mechanism of action is the inhibition of DNA gyrase and topoisomerase IV, enhancing the Gram-positive spectrum, especially for ocular infections [73].

Sitafloxacin belongs to this generation. It was labelled with ^99m^Tc and biodistribution studies and scintigraphic images were evaluated, respectively in rats and rabbits, where infection was induced with 2 × 10^8^*S. aureus* and inflammation induced with turpentine oil. Biodistribution confirmed the renal excretion also for this class of antibiotics with a high accumulation in infected muscles confirmed by in vivo images and* T*/NT ratio equal to 23.13 ± 0.1 at 2 h post injection. This* T*/NT ratio was the highest obtained with a radiolabelled antibiotic suggesting sitafloxacin as the best imaging agent for imaging infections caused by *S. aureus* [74]. It would be important to determine whether it can image also other strains of bacteria and whether the accumulation lasts over time.

Due to initial enthusiasm, sitafloxacin was chemically modified to sitafloxacin-dithiocarbamate, which is more stable, and then labelled with ^99m^Tc via a [^99m^TcN]^2+^ core. Biodistribution studies and whole body images were performed in rats and rabbits, infected with *S. aureus* and turpentine oil and heat killed bacteria as controls. Experimental data showed a clearance through the kidneys and confirmed the high uptake in the infected muscle with living bacteria. The* T*/NT ratio was 7.40 ± 1 after 2 h from injection in the infectious foci, as compared to 1 ± 1 in the inflamed area, confirming this radiopharmaceutical as a very promising infection imaging agent [75].

^99m^Tc-moxifloxacin could be considered another potential agent. It was studied in rats and rabbits after the induction of a septic inflammation with *E. coli* in the thigh muscle. On images it was possible to notice the infected site in a clear way, with a specific accumulation six times higher than in normal tissues [76].

Another antibiotic of this generation, specific for *S. pneumoniae* infection, is gemifloxacin. After labelling with ^99m^Tc, it was tested in infected, inflamed and normal rats. Results showed an early uptake in the liver, followed by a renal clearance; the* T*/NT ratio between infected and normal muscle was maximum at 90 min and then decreased slightly [77]. Recently, another study, performed by Shahzad et al. [78], confirmed the specificity of ^99m^Tc-gemifloxacin to localize respiratory tract infections. The radiopharmaceutical was studied in rabbits infected with three different strains of bacteria (3 × 10^8^ CFU), including *K. pneumoniae*, *S. typhi* and *P. aeruginosa*. The maximum* T*/NT ratios were 8, 8.87 and 16.5 at 4 h post-injection, respectively for the three kinds of bacteria, confirming that ^99m^Tc-gemifloxacin could be used as a bacterial imaging agent for lung infections.

Finally, another fluoroquinolone derivative has been proposed as ciprofloxacin’s substitute by Moustapha et al. [79]. ^99m^Tc-sarafloxacin was studied in vitro and in *S. aureus* infected mice, while as turpentine oil and heat killed bacteria were used to induce the aseptic inflammation. Experimental data revealed both renal and hepatic excretion with a low uptake in the infectious foci as compared to other quinolones of fourth generation.* T*/NT ratio in infected mice was 4.2 ± 0.1 at 2 h post injection, versus 3.4 and 3.3 for turpentine oil and heat killed bacteria.

## Cephalosporins

Cephalosporins have also been radiolabelled for bacteria imaging in vivo. In 2013 El-Tawoosy et al. studied the best labelling condition of cephazolin with ^99m^Tc and its biological distribution in murine model, infected with *S. aureus* (10^7^–10^8^ CFU) and turpentine oil as control. Results showed a good preparation and labelling of the product, a rapid distribution in mice with excretion through kidneys and intestine by 2 h, and a infected/inflamed muscle ratio (*T*/NT) equal to 4.60 ± 0.21 at 2 h. However, since the highest ratio was 8.57 ± 0.40 at 30 min, cephazolin is able to distinguish well the early stages of infection from sterile inflammation [80].

The second generation of cephalosporins has a spectrum of activity like the first generation antibiotics, but more active against Gram-negative bacteria, and includes antibiotics as cefuroxime axetil, whose bactericidal activity is the inhibition of cell wall synthesis through the binding to specific proteins. Its potential use as a radiopharmaceutical has been tested in rats with sterile and septic inflammation, caused by 10^8^ CFU of *S. aureus*, in the Yurt Lambrecht’s study. Results showed a rapid clearance by liver and kidney and a better retention in infectious areas than sterile inflamed areas because of its specific binding to gyrase enzymes. However, authors reported a low* T*/NT ratio at 30 min (1.6), with a slight increase at 4 h (2.5). This suggests that ^99m^Tc-cefuroxime acetil could be a promising infection imaging agent, but more studies are needed to confirm this hypothesis [81]. Cefuroxime is another second-generation cephalosporin antibiotic that was labelled and tested in a study performed by Chattopadhyay et al. [82]. After labelling with ^99m^Tc, the compound was injected in rats infected with 10^6^–10^8^ CFU of *E. coli* bacteria in the left thigh. Experimental data showed a renal and hepatic excretion and a poor accumulation in the infection site, confirmed by the* T*/NT ratio (1.8) at 3 h from the injection. Therefore ^99m^Tc-cefuroxime is not entirely able to distinguish bacterial infections. Third-generation cephalosporins are broad-spectrum antimicrobial agents used in many clinical situations. Among them, ceftizoxime has the best Gram-positive coverage [83]. Gomes Barreto et al. labelled it with ^99m^Tc for imaging of *E. coli* infection in rats’ muscle compared to controls and animals bearing a sterile zymosan induced abscess. Experimental data underlined a maximum uptake in kidneys and a significant uptake in the septic muscle rather than in the sterile one. The uptake persisted up to 6 h, as confirmed by a* T*/NT ratio of 3.24 ± 1 in the infection site, (1.65 ± 0.23 in controls). On the basis of obtained data, ^99m^Tc-ceftizoxime showed a moderate specificity that lead researchers to investigate its use in other models [84].

Costa et al. tested this radiolabelled antibiotic for the diagnosis of deep sternal wound infection. They used twenty rats divided into four groups, two controls and two with sternotomy and infection with *S. aureus*. Scintigraphic images revealed a higher levels of radioactivity, expressed as number of counts, in the region of interest of infected rats (12,258.2 ± 1729 counts/10 min) than control counterparts (4920.6 ± 562.9) in different time points after injection. This result confirmed that ^99m^Tc-ceftizoxime is a potential antimicrobial agent, which detects infection post sternotomy [85].

Also Teixeira et al. [86] used ^99m^Tc-ceftizoxime for the diagnosis of suspected infections in titanium implants in rat model. Control rats received a sterile implant, while experimental group received an implant infected with 10^9^ CFU of *S. aureus*. Scintigraphic images showed higher uptake in infectious area in rats than in controls, expressed as the difference between groups, at 6.5 h post-injection. Despite these promising results in localizing infected implants, further studies are required to improve sensitivity and specificity of ^99m^Tc-ceftizoxime.

Cefotaxime has a similar structure of ceftizoxime and was studied by Mirshojaei et al. [87] as a potential infection-imaging agent. After labelling with ^99m^Tc, the biological distribution was performed in mice, infected with 10^8^ CFU of *S. aureus* bacteria in the thigh muscle. Results showed a renal clearance, low hepato-biliary excretion and a poor accumulation in the infectious site with the maximum* T*/NT ratio at 1 h (2.89 ± 0.58). Although a more rapid metabolic route, when compared to ^99m^Tc-ciprofloxacin, ^99m^Tc-cefotaxime requires more studies to demonstrate its specificity. Ilem-Ozdemir and coll. [88] labelled with ^99m^Tc the cefotaxime sodium. Then, they evaluated its biodistribution in rats, infected with 4 × 10^10^ CFU of *E. coli* or turpentine oil as control. Results showed a main renal excretion of radiopharmaceutical and a very poorly uptake in the infectious foci. Indeed the* T*/NT ratios were 3.77 ± 2.38 and 3.30 ± 0.94 at 1 h post injection.

Another third-generation cephalosporin, tested by various authors, is ceftriaxone. Also for this antibiotic, similar results were obtained and ^99m^Tc-ceftriaxone could be able to distinguish sterile and septic inflammation. The first study, performed by Mostafa et al. in 2010, describes the labelling of ceftriaxone with ^99m^Tc and its biodistribution in a mouse model, infected with alive *E. coli*, heat killed bacteria and turpentine oil as controls. In this study the ability to differentiate between bacterial infection and sterile inflammation was demonstrated in vitro and confirmed in vivo. In mice, it showed renal excretion and a good retention at the infectious site because of its specific binding to bacteria.* T*/NT ratio for the living bacteria was 5.67 ± 0.6 at 4 h post injection as compared to the turpentine oil and heat killed *E. coli* ratios that were less of 2 [89]. The second study about ceftriaxone was published by Kaul et al. in 2012. The main purpose of the study was to assess the efficacy of ^99m^Tc-ceftriaxone in vitro through bacterial binding assay with living and heat killed *S. Aureus*, but also in vivo in murine and rabbit models and in humans. Results confirmed the ability of the labelled antibiotic to discriminate between inflammation and infection: in fact scintigraphic images in rabbit showed a higher uptake in the infectious site than in the inflamed muscle at 4 and 24 h, and also the* T*/NT ratio in mice with septic lesion was 4.5 at 24 h as compared to sterile inflammation that showed 1.4 at 24 h. Clinical studies demonstrated that the radiolabelled antibiotic localizes acute bacterial infections, especially in bacterial osteomyelitis and could be used for diagnosis of other orthopaedic infections too [90]. A third study with ^99m^Tc-ceftriaxone was performed by Fazli et al. [91], but it did not confirm the good specificity previously published by others. They tested it in a murine model, comparing an infection with living *S. aureus*, to a sterile inflammation with heat killed bacteria or turpentine oil. Experimental data showed a renal excretion and a poorly specific accumulation in the infected muscle in comparison to inflamed and normal muscles. The *T*/NT ratio in infected muscles was 3.39 ± 0.6 at 3 h post injection, while the *T*/NT in muscles with turpentine oil or with heat killed bacteria were, respectively, 3.12 ± 0.35 and 2.48 ± 0.45 always at 3 h post injection with no statistically significant difference between the 3 groups [91]. Finally, Sohaib et al. [92] confirmed the ability of this radiopharmaceutical to discriminate the infection from inflammation. ^99m^Tc-ceftriaxone was tested in rats, infected with 10^8^ CFU of *S. aureus* or *E. coli*, whereas turpentine oil was used in control rats. Biodistribution studies revealed a main renal excretion, followed by liver and intestine, and high accumulation in the infectious area in animals injected with *E. coli* rather than *S. aureus* or turpentine oil. These data were confirmed by* T*/NT ratios equal to 12.66 ± 1.44, 2.35 ± 0.21 and 1.4 ± 0.01, respectively, suggesting that ^99m^Tc-ceftriaxone could be used as a microbial imaging agent only for *E. coli*.

Another third-generation antibiotic, studied by Mirshojaei et al. is ceftazimide. It was labelled with ^99m^Tc and its biodistribution was tested in normal and *S. aureus* infected mice. Data showed a similar uptake of radiopharmaceutical in non target organs (liver, spleen, heart and lung) between control and infected animals with lower hepato-biliary excretion when compared to ^99m^Tc-ciprofloxacin; about accumulation in the infected and control muscle, the ratio was 1.4 ± 0.2 at 1 h post injection and 1.1 ± 0.1 at 4 h. Therefore, ceftazimide did not show the same specificity of ceftizoxime and ceftriaxone, as bacterial imaging agent [93].

Cefoperazone is another third-generation cephalosporine, studied to evaluate the best radiolabelling conditions with ^99m^Tc and its biological distribution in a rat model of *S. aureus* bacterial infection. In vivo results, expressed as  %ID/g, showed a renal clearance and a 4.5-fold higher uptake in the infected tissue than control, with a maximum* T*/NT ratio at 45 min post injection of 4.66 ± 0.53: then this value decreased with time (2.9 ± 0.75 at 5 h), probably because of bacterial killing by radiopharmaceutical or clearance from circulation. These data make cefoperazone a promising agent for detection of infectious foci, even if it needs further investigations [94].

Belonging to fourth-generation of cephalosporins is cefepime, whose biological efficacy and specificity were compared to gatifloxacin, a fluoroquinolone derivative. The two radiopharmaceuticals were labelled with ^99m^Tc and tested in rats infected with living *E. coli*, heat killed bacteria and turpentine oil. After successful in vitro quality controls and bacterial binding assay, biodistribution studies were performed and results demonstrated a liver uptake for both radiopharmaceuticals that decreases with time. The uptake in the infectious foci was better for ^99m^Tc-cefepime than for ^99m^Tc-gatifloxacin (*T*/NT ratio was 8.4 ± 0.1 at 3 h post injection for ^99m^Tc-cefepime and 4.5 ± 0.3 for ^99m^Tc-gatifloxacin in infected muscles with living bacteria): Thus, cefepime was able to distinguish between sterile and septic inflammation better than all other antibiotics [95].

## Inhibitors of nucleic acid synthesis

The inhibition of nucleic acid synthesis occurs through the binding of the antimicrobial to DNA-dependent RNA polymerase, blocking the initiation of RNA synthesis, or to DNA gyrase, inhibiting DNA synthesis [96].

Rifampicin is particularly indicated for the treatment of tuberculosis, and recently an imaging agent for PET use has been developed for latent tuberculosis detection, labelled with ^11^C. ^11^C-rifampicin was tested in preclinical studies to evaluate whether there is sufficient drug in the infected site because the radiopharmaceutical is able to accumulate in a hypoxic environment like the tuberculotic granuloma [97].

However, in animals rifampicin was studied for detection of methicillin-resistant *S. aureus* (MRSA) infections in both rats and rabbits. Turpentine oil induced inflammation is always the method of choice for control. After labelling with ^99m^Tc, biodistribution revealed a long renal clearance, and a high accumulation in the infectious foci, confirmed by in vivo calculated* T*/NT ratio (7.34 ± 0.74 at 90 min post injection) [98].

Another antibiotic that indirectly acts on nucleic acid, in particular DNA, is nitrofurantoin: it is often used for urinary tract infections because many uropathogens have not yet developed resistance to it. Its mechanism of action is still unclear, but it seems that bacterial nitroreductase enzymes transform the antibiotic into more reactive intermediates that lead to single-strand breaks in DNA through interaction with bacterial ribosomal proteins [99]. ^99m^Tc-nitrofurantoin was investigated in *E. coli* infected rats and rabbits. In vivo distribution showed an early uptake in the liver and stomach, while the accumulation in infectious foci rapidly increased in a time-dependent manner as compared to controls, with a peak at 90 min p.i., with a* T*/NT ratio equal to 4.83 ± 1.13 [100].

## Inhibitors of bacterial cell wall synthesis

This category of antibiotics may inhibit many steps of cell wall synthesis, above all the inhibition of peptidoglycan synthesis, because cell wall is essential for survival of bacteria; but also the membrane transport mechanisms, resulting in osmotic lysis [101].

An example of these antibiotics is the well-known amoxicillin, a penicillin derivative that acts by inhibiting the third and last stage of bacterial cell wall synthesis. It is particularly active on *S. pneumoniae* [102]. Amoxicillin was recently labelled with ^99m^Tc and its biological distribution was studied in *S. pneumoniae* infected rabbits. Results were promising but not as good as for other radiolabeled antibiotics and maximum accumulation in the infection was recorded 2 h post-injection [103].

By contrast, alafosfalin is a dipeptide phosphonic acid, active against both Gram-positive and Gram-negative bacteria. It inhibits the early stage of peptidoglycan synthesis because it mimics the terminal dipeptide moiety (D-Ala-D-Ala), inhibiting the enzyme D-Ala-D-Ala synthetase, or inhibits the enzyme alanine racemase for its affinity to racemase cofactors [104]. When labelled with ^99m^Tc it showed rapid renal excretion in rats, infected with 10^8^ CFU of *S. Aureus*. Interestingly, Tsopelas et al. compared ^99m^Tc-alafosfalin with ^99m^Tc-DTPA and ^99m^Tc-labelled-leukocytes and showed that the* T*/NT ratio at 4 h p.i. for ^99m^Tc-alafosfalin was higher than for ^99m^Tc-DTPA (4.32 ± 0.26 vs 1.93 ± 0.15) but lower than for ^99m^Tc-WBC. These results were also confirmed by scintigraphic images and histological studies, suggesting that ^99m^Tc-alafosfalin complex is not as specific as WBC for detecting bone infections, particularly in case of high probability of infection [105].

The bacterial cell wall is mainly composed by peptidoglycan, which is formed from alternating units of *N*-acetylglucosamine and *N*-acetylmuramic acid. The introduction of positron emitter isotope into *N*-acetylglucosamine structure could be a solution for the detection bacteria using PET imaging [5]. Thus, Martìnez et al. described a new labelling method of 2-deoxy-2-[^18^F]fluoroacetamido-D-glucopyranose ([^18^F]FAG) trough microwave irradiation, and demonstrated its ability to discriminate, in vivo, a bacterial infection from a sterile inflammation. They used a mouse model, infected with 10^7^ CFU of *E. coli* or a sterile inflammation with turpentine oil for biodistribution studies and rats for acquiring PET images, followed by histology and immunostaining of relevant tissues. Images showed a high accumulation of [^18^F]FAG in the infectious foci, similar to [^18^F]FDG, but there was no uptake of [^18^F]FAG in the sterile inflammatory lesion as compared to [^18^F]FDG. Haematoxylin-eosin and immunostaining using anti-*E. coli* antibodies confirmed the presence of bacteria in the infected tissue and an infiltration of granulocytes and macrophages, while in turpentine oil-induced inflammation, neutrophils and macrophages prevailed, demonstrating that [^18^F]FAG is able to distinguish bacterial infections from inflammation in contrast to [^18^F]FDG [106].

Another antibiotic that inhibits the bacterial cell wall synthesis is vancomycin. Because of its big size and complex structure, vancomycin does not enter the membrane of Gram-negative bacteria, but binds to peptidoglycan precursors, preventing their lipid carrier-mediated transfer through the membrane [107]. Vancomycin was also labelled with ^99m^Tc and in vitro studies (binding assay to bacteria and stability test) were performed as well as in vivo studies (biodistribution and targeting in *S. aureus* infected rats). Results showed both liver and kidneys metabolism and a high uptake of in the infected muscle with a* T*/NT ratio equal to 5 at 60 min post injection [108].

## Inhibitors of protein synthesis

Protein synthesis inhibitors include various classes of antibiotics, each of which blocks the process in a different way, in particular at the ribosomal level [109].

An example is kanamycin, a bactericidal agent of aminoglycoside family, used for the treatment of infections when penicillin cannot be used such as bone, skin or abdominal infections. Its mechanism of action is the premature chain termination and RNA codon misreading by the interference with 30S ribosome. It was labelled with ^99m^Tc by a simple and easy procedure and then tested in rats for in vivo distribution and in rabbits for scintigraphy, in which infection was induced with 2 × 10^8^ CFU of *S. aureus*. The tissue distribution showed a renal elimination and a high uptake in the infectious foci as compared to normal muscle used as control, with a* T*/NT ratio greater than 2 up to 24 h from injection [110].

Belonging to these inhibitors there are two other antibiotics, doxycycline hyclate (DOX) and erythromycin. DOX is an antibacterial tetracycline derivative, with a wide range of activity against Gram-negative and Gram-positive bacteria; it binds to 30S subunit of ribosome, preventing the binding between aminoacyl tRNA and the acceptor site on mRNA. ^99m^Tc-DOX was tested in vivo in rats, infected with 4 × 10^10^ CFU of *E. Coli*. The excretion was mainly through kidneys, but also through stomach because of high intestinal activity despite liver uptake was low. The highest* T*/NT ratio was 2.62 ± 0.88 after 5 h from the radiotracer injection. According to previous studies, the radiopharmaceutical had a high uptake both in the infected and inflamed thigh muscle, indicating that ^99m^Tc-DOX cannot differentiate bacterial infection from sterile inflammation [111].

Erythromycin is a bacteriostatic agent of macrolides family and it inhibits the transpeptidation or translocation because of a missed binding of tRNA to the specific site by the binding to 50S ribosomal subunit [96]. Biodistribution studies were performed in mice infected with 10^5^–10^6^ CFU of *S. aureus* or turpentine oil as control. Experimental data showed a main elimination through renal and urinary pathway at 4 h from injection of radiotracer and a liver uptake that decreased with time. The* T*/NT ratio of ^99m^Tc-erythromycin in infected muscle was greater than ciprofloxacin (5 ± 0.6 vs 3.8 ± 0.8) at 30 min post injection, but at the same time values of* T*/NT ratio were comparable in infected and inflamed mice, respectively 5 ± 0.6 and 4.8 ± 0.4. Thus, ^99m^Tc-erythromycin complex accumulates in infected muscles, but it cannot distinguish between septic and aseptic inflammation [112]. Another not very specific antibiotic of this category is vibramycin. It was labelled with ^99m^Tc and then tested in a rats. The infection was induced with 2 × 10^8^ CFU of live *S. aureus*, while for the inflammation heat-killed bacteria or turpentine oil were used. Biodistribution revealed a main hepato-biliary excretion and not high accumulation of radiopharmaceutical in the infectious site compared to controls, confirmed by similar values of* T*/NT ratios (2.64, 2.15 and 1.80, respectively in live bacteria, heat killed bacteria and turpentine oil). Therefore these results show that ^99m^Tc-vibramycin cannot be considered a specific infection imaging agent [113].

By contrast, azithromycin, clarithromycin and clindamycin are three inhibitors of protein synthesis, which could be novel potential bacterial imaging agents. Azithromycin, like erythromycin, belongs to macrolides, but differs for the structure and the activity level against Gram-positive and Gram-negative bacteria [114]. It was also labelled with ^99m^Tc and biodistribution studies were performed in mice, where infection was induced with *S. aureus* in the thigh muscle. Inflammation was induced with direct injection of turpentine oil and heat killed bacteria. The quantitative evaluation, expressed as the percentage of injected dose per organ, showed an excretion through kidneys and urine, and high accumulation in infectious muscle than controls, confirmed by the* T*/NT ratio: the maximum peak was 6.20 ± 0.12 at 2 h post-injection, but at all time intervals values were significantly higher than sterile inflamed muscles [115].

Clarithromycin is a derivative of erythromycin and it was labelled with ^99m^Tc. Mice infected with 10^8^ CFU of *S. aureus* were used as a model, while turpentine oil and heat killed bacteria were used as control. Biodistribution showed an excretion of radiopharmaceutical mainly through the urinary pathway and a high uptake in the site of infection was observed as compared to controls.* T*/NT ratios were 7.33 ± 0.13 at 2 h for the infection model, while 3.1 ± 0.13 and 3.26 ± 0.12 for turpentine oil and heat killed bacteria, confirming the ability of ^99m^Tc-clarithromycin to distinguish between septic and sterile inflammation [116].

Clindamycin is an antibiotic of lincosamide family, used for treatment of streptococci and staphylococci infections. It binds to the 23S rRNA of the 50S ribosomal subunit, inhibiting the initial stage of the elongation cycle during protein synthesis [117]. After labelling with ^99m^Tc, in vivo distribution and scintigraphic imaging were performed, respectively in rats and rabbits. The infection was induced using 2 × 10^8^ CFU of *S. aureus*, while inflammation with turpentine oil and heat killed bacteria. ^99m^Tc-clindamycin was eliminated through kidneys and it mostly accumulated in the infectious foci as compared to inflamed muscles, indicating a specific binding to living bacteria. However, the* T*/NT ratio was not very high, since it was 3.1 ± 0.3 after 1 h post-injection [118].

## Others

Mebendazole is an anthelmintic drug with a broad spectrum against nematodal and cestodal species; it belongs to the imidazole group and it is particularly indicated for the treatment of trichinellosis [119]. In fact, in the study performed by Inceboz et al. [120], the authors wanted to investigate the biodistribution of ^99m^Tc-mebendazole in a rat model, infected with *T. spiralis*, a nematode that is often present in wild carnivorous animals. Briefly, 750–1000 larvae were orally administrated in rats to induce infection in the muscles, while healthy rats were used as controls. Once the infection was established, ^99m^Tc-mebendazole was given to rats by oral administration or through a tail vein. Biodistribution data showed a main uptake in the gastro-intestinal tract, if the administration was oral, while in kidney if it was injected i.v. The maximum uptake in muscles was found in the tongue and the diaphragm for both groups, but also in other infected muscles such as masseter or semimembranosus muscle, suggesting that ^99m^Tc-mebendazole complex could be a useful imaging agent to detect *T. spiralis* infections.

Fluoromaltose is another molecule through which it is possible to distinguish bacterial infections in vivo from other pathologies. In this case maltodextrin-based imaging probes (MDPs) were used, exploiting a bacteria-specific mechanism of transport, called maltodextrin transporter, which is absent in mammalian cells. These probes were internalized only by bacteria with a rapid metabolism with high sensitivity, detecting low number or bacteria and discriminating between infection and inflammation [121]. Based on these considerations, Gowrishankar et al. [122] labelled 6-fluoromaltose with ^18^F to evaluate its ability to differentiate bacterial infection from inflammation in a murine model. Infection was induced with 5 × 10^7^ CFU of *E. coli*, while the inflammation was produced with 10^8^ CFU of heat-killed bacteria and turpentine oil. Micro PET/CT images were acquired as well as biodistribution studies and histology. A 3D color map from PET/CT images showed a clear accumulation of 6-[^18^F]-fluoromaltose in the infected muscle compared to non infected muscle and a renal and hepatobiliary excretion, confirmed by biodistribution, histological images and bioluminescence imaging.

Triacetylfusarinine C (TAFC) and ferrioxamine E (FOXE) are two siderophores, which are produced by various microorganisms for the binding and storage of iron. Indeed iron is essential for many metabolic processes of microorganisms. In biofilms specific transporters for ^68^Ga-siderophores are upregulated, resulting in an accumulation of the radiopharmaceutical in bacteria. Considering the similar chemistry of iron and gallium, Petrik et al. [123, 124] investigated the possibility to label TAFC and FOXE with ^68^Ga and then they evaluated the capacity of radiopharmaceuticals to localize infection by *A. fumigatus* in a rat model. In vitro studies were also performed and included a comparison of uptake between different bacteria (*A. fumigatus*, *P. aeruginosa*, *S. aureus*) and human lung cancer cells. In vivo studies showed a rapid accumulation of ^68^Ga-TAFC and ^68^Ga-FOXE in *A. fumigatus* infected tissues, especially in lungs, while a moderate uptake in the turpentine oil inflamed muscle and no uptake in *S. aureus* infected muscle was observed. These data support the conclusion that ^68^Ga-TAFC and ^68^Ga-FOXE are selective agents to detect *A. fumigatus* infection through PET imaging, with a higher sensitivity for ^68^Ga-FOXE.

Finally, 2,2′-[(8-hydroxyquinolin-7-yl) methylazanediyl] diacetic acid (HQMADA) is an antibacterial drug, deriving from the reaction between 8-hydroxyquinoline and iminodiacetic acid in presence of paraformaldehyde. It was labelled with ^99m^Tc and, after in vitro studies such as stability in serum and binding to bacteria, biodistribution was studied in *E. coli* mice. Experimental data revealed a main uptake in liver and intestine and a high accumulation in the infectious foci than in sterile inflammation.* T*/NT ratio was 5.52 ± 0.2 between infected and healthy muscle after 2 h from injection, while in the inflamed model, both with turpentine oil and heat-killed *E. coli*, the* T*/NT ratios were nearly 2 at each time point, suggesting that ^99m^Tc-HQMADA complex can differentiate bacterial infection from sterile inflammation [125].

## Summary of systematic analysis of the literature

The systematic analysis was performed by searching in PubMed, Web of Science, Scopus and Google Scholar websites, for “radiolabelled OR radiolabeled OR labelled OR labeled AND antibiotic* AND bacteria*”. We obtained 1193 papers from PubMed of which 25 original articles were considered, and eight reviews, one case report and one editorial were excluded. These papers were integrated with similar search in other websites, finally obtaining 81 original published studies that were analysed and included in this systematic review and summarized in Tables 1 and 2. We considered: the type of isotope, the labelling method, the specific activity of radiopharmaceutical, its stability in serum and/or saline, the animal model used, the metabolic route, the control experiment and the obtained results in terms of target muscle/background (*T*/NT) ratio, with the purpose of having an objective analysis as complete as possible.

**Table 1:** Comparative analysis of paper published with radiolabelled ciprofloxacin in humans and animals

First author (ref.)	Antibiotic	Isotope	Labelling method	Specific activity (MBq/mmol)	Stability		Model of study	Metabolic route
					Saline	Serum		
Brunner [13]	Ciprofloxacin	^18^F	–	0.4	–	–	Healthy volunteers	PK: low in SNC, liver and kidneys
Langer [27]	Ciprofloxacin	^18^F	–	0.342	–	–	4 patients	–
Dumarey [51]	Ciprofloxacin	^99m^Tc	Stannous tartrate	6.33 × 10^4^	–	–	30 patients	Liver and kidneys
De Winter [30]	Ciprofloxacin	^99m^Tc	Stannous tartrate	6.33 × 10^4^	–	–	6 volunteers	Liver (+), kidneys (++)
De Winter [52]	Ciprofloxacin	^99m^Tc	–	–	–	–	48 patients	–
Appelboom [56]	Ciprofloxacin	^99m^Tc	–	–	–	–	86 patients	Liver (+), kidneys (++)
Falagas [31]	Ciprofloxacin	^99m^Tc	Stannous tartrate	–	–	–	11 patients	–
Amaral [38]	Ciprofloxacin	^99m^Tc	FSA	6.53 × 10^4^	98 % at 8 h	–	3 patients	Liver (+), kidneys (++)
Dutta [48]	Ciprofloxacin	^99m^Tc	–	–	–	–	25 diabetic patients (foot ulcers)	–
Hall [32]	Ciprofloxacin	^99m^Tc	FSA	–	–	–	90 patients	Liver (+), kidneys (++)
Sharma [43]	Ciprofloxacin	^99m^Tc	Stannous tartrate	9 × 10^4^	–	–	21 patients	–
Fuster [41]	Ciprofloxacin	^99m^Tc	Tartaric acid	–	–	–	40 patients	–
Larikka [42]	Ciprofloxacin	^99m^Tc	–	–	–	–	16 patients	–
Sharma [44]	Ciprofloxacin	^99m^Tc	Stannous tartrate	9 × 10^4^	–	–	22 patients	–
Choe [40]	Ciprofloxacin	^99m^Tc	Stannous tartrate	9 × 10^4^	–	–	16 patients	Liver (+), kidneys (++)
Britton [28]	Ciprofloxacin	^99m^Tc	FSA	6.53 × 10^4^	98 % at 8 h	–	99 patients	Liver (+), kidneys (++)
Malamitsi [39]	Ciprofloxacin	^99m^Tc	–	–	–	–	33 patients	–
Britton [33]	Ciprofloxacin	^99m^Tc	Stannous tartrate	5.85 × 10^4^	–	–	879 patients	Liver (+), kidneys (++)
Bhardwaj [50]	Ciprofloxacin	^99m^Tc	SnCl_2_	–	–	–	25 patients	Liver (+), kidneys (++)
Sonmezoglu [46]	Ciprofloxacin	^99m^Tc	Stannous tartrate	6.13 × 10^4^	–	–	56 patients	Liver (+), kidneys (++)
Gallowitsch [49]	Ciprofloxacin	^99m^Tc	–	–	–	–	20 patients	–
Obradovic [37]	Ciprofloxacin	^99m^Tc	SnCl_2_	–	–	–	27 patients	Liver (++), kidneys (+)
Artiko [36]	Ciprofloxacin	^99m^Tc	SnCl_2_	–	–	–	21 patients	Liver (++), kidneys (+)
Sarda [53]	Ciprofloxacin	^99m^Tc	Stannous tartrate	6.40 × 10^4^	–	–	16 patients	Liver (+), kidneys (++)
Vinjamuri [8]	Ciprofloxacin	^99m^Tc	FSA	1.63 × 10^5^	98 % at 8 h	–	56 patients	Liver (+), kidneys (++)
Larikka [34]	Ciprofloxacin	^99m^Tc	Stannous tartrate	–	–	–	30 patients	Liver (+), kidneys (++)
Gemmel [55]	Ciprofloxacin	^99m^Tc	–	1.63 × 10^5^ (Vinjamuri)	98 % at 8 h	–	22 patients	–
Singh [45]	Ciprofloxacin	^99m^Tc	Stannous tartrate	1.19 × 10^5^	94.85 % at 24 h	–	77 patients	Liver (+), kidneys (++)
Malamitsi [47]	Ciprofloxacin	^99m^Tc	Stannous tartrate	6.5 × 10^4^	–	–	45 patients	–
Lee [35]	Ciprofloxacin	^99m^Tc	Stannous tartrate	9.06 × 10^4^	–	–	21 participants	–
Pucar [54]	Ciprofloxacin	^99m^Tc	–	–	–	–	40 patients	–
Satpati [18]	Ciprofloxacin conjugates	^68^Ga	DOTA (1) /NOTA (2)	6.2 × 10^6^	90/98 % at 4 h	85/90 % at 4 h	Rat	Liver (+), kidneys (++)
Oh [16]	Ciprofloxacin	^99m^Tc	FSA/SnCl_2_	1.77 × 10^5^/1.75 × 10^5^	90 % at 6 h	80 % at 6 h	Rat	Kidneys
Doroudi [17]	Ciprofloxacin	^99m^Tc	–	–	–	–	Rat	–
Aungurarat [19]	Ciprofloxacin	^99m^Tc	SnCl_2_	1.65 × 10^5^	90 % at 6 h	–	Mouse	–
Mirshojaei [22]	Ciprofloxacin	^99m^Tc	SnCl_2_	1.78 × 10^5^	–	84.2 % at 1 h	Mouse	Liver (++), kidneys (+)
Zhang [20]	Ciprofloxacin	^99m^Tc	Stannous tartrate	2.77 × 10^4^	90 % at 6 h	–	Mouse	Liver (++), kidneys (+)
Bhardwaj [14]	Ciprofloxacin	^99m^Tc	Stannous tartrate	1.10 × 10^4^	94.85 % at 24 h	–	Mouse (biodis)/rabbit (imaging)	Liver (+), kidneys (++), intestine (+)
Sarda [23]	Ciprofloxacin	^99m^Tc	Stannous tartrate	6.40 × 10^4^	–	31 % at 4 h	Rabbit	Liver (+), kidneys (++)
Peremans [26]	Ciprofloxacin	^99m^Tc	–	–	–	–	Dog	–
Alexander [25]	Ciprofloxacin	^99m^Tc	SnCl_2_	1.46 × 10^5^	–	–	5 camelids, goat	Liver (+), kidneys (++), lungs
Wang [24]	Ciprofloxacin	^99m^Tc	Stannous tartrate	5.55 × 10^4^	90 % at 6 h	–	27 Swine	Liver and kidneys
Dahiya [21]	Ciprofloxacin and derivatives	^99m^Tc	Stannous tartrate/SnCl_2_	–	90 % at 24 h	–	Mouse	Liver (++), kidneys (+)
Zhang [57]	Ciprofloxacin dithiocarbamate	^99m^Tc	Direct labelling—SnCl_2_	–	95 % at 6 h	–	Mouse	Liver (++), kidneys (+)
Zhang [58]	Ciprofloxacin dithiocarbamate	^99m^Tc	[^99m^Tc(CO)_3_(H_2_O)_3_]+	16.82–336.4	95 % at 6 h	80 % at 3 h	Mouse	Liver, lung, spleen

First author (ref.)	Max* T*/NT ratio						Control experiment	Imaging method	Comment by authors
	Time	Bacteria (CFU—strain)	Infection site	BKG	*T*/NT infection	*T*/NT control			
Brunner [13]	–	–	–	–	–	–	–	PET	Safe and useful
Langer [27]	–	–	–	–	–	–	–	PET	Non specific binding to bacteria in vitro and in vivo
Dumarey [51]	–	–	–	–	–	–	41 healthy subjects	Scintigraphy	Sensitive but not specific
De Winter [30]	–	–	–	–	–	–	–	Whole body scan	Favourable for clinical SPECT imaging
De Winter [52]	–	–	–	–	–	–	–	SPECT/planar imaging	Better sensitivity SPECT but less specificity for postoperative spine
Appelboom [56]	–	–	–	–	–	–	20 healthy subjects	Scintigraphy	Not specific for infections, but promising for joint inflammations
Falagas [31]	–	–	–	–	–	–	–	Scintigraphy	Useful in the diagnosis of active spinal infections, but needs further studies
Amaral [38]	–	–	–	–	–	–	^99m^Tc-HMPAO	Scintigraphy	Better than HMPAO for the diagnosis of osteomyelitis of the axial skeleton
Dutta [48]	–	–	–	–	–	–	–	Scintigraphy	Specificity and sensitivity improve with bone scan
Hall [32]	–	–	–	–	–	–	–	–	Sensitive and specific
Sharma [43]	–	–	–	–	–	–	Ultrasound	Scintigraphy	Useful in the detection of pelvic inflammatory disease (PID)
Fuster [41]	–	–	–	–	–	–	Leukocyte and bone marrow scintigraphy (LS-MS)	Scintigraphy	Sensitive and specific for hip prosthesis infections, but not for knee prosthesis
Larikka [42]	–	–	–	–	–	–	^99m^Tc-leukocyte	Scintigraphy	Valid alternative to 99mTc-leukocyte for knee prosthesis diagnosis
Sharma [44]	–	–	–	–	–	–	^99m^Tc-MDP	Scintigraphy	Useful in the detection of tubercolar bone disease
Choe [40]	–	–	–	–	–	–	–	SPECT	Sensitive and specific for the diagnosis of acute cholecystitis
Britton [28]	–	–	–	–	–	–	^111^In-WBC/^99m^Tc-WBC	Scintigraphy	Effective and specific when there is a suspected infection
Malamitsi [39]	–	–	–	–	–	–	Erythrocyte sedimentation rate, C-reactive protein	–	Sensitive and accurate for chronic bone and joint infections
Britton [33]	–	–	–	–	–	–	–	Scintigraphy	Sensitive and specific
Bhardwaj [50]	–	–	–	–	–	–	–	Scintigraphy	Promising agent for osteoarticular tubercolosis
Sonmezoglu [46]	–	–	–	–	–	–	^99m^Tc-WBC	Scintigraphy	Better in spinal infections, potential agent
Gallowitsch [49]	–	–	–	–	–	–	^99m^Tc-mAb anti-granulocyte	Immunoscintigraphy	Potential infection imaging agent
Obradovic [37]	–	–	–	–	–	–	–	Scintigraphy	Sensitive and specific method to detect early imaging of orthopaedic infections
Artiko [36]	–	–	–	–	–	–	–	Liver/spleen scintigraphy	Useful to detect suspected abdominal and gastrointestinal infections
Sarda [53]	–	–	–	–	–	–	–	Scintigraphy	Good sensitivity, negative predictive value, no discrimination (bone inf)
Vinjamuri [8]	–	–	–	–	–	–	^111^In-WBC	Scintigraphy	High specificity and support to radiolabelled WBC
Larikka [34]	–	–	–	–	–	–	^99m^Tc-leukocyte	Scintigraphy	Suitable for diagnosis of hip prosthesis infections
Gemmel [55]	–	–	–	–	–	–	–	SPECT/planar imaging	Better in spinal infections, limited specificity, non specific for postoperative spine
Singh [45]	–	–	–	–	–	–	–	Scintigraphy	To increase the confidence analysis after 99mTc-MDP
Malamitsi [47]	–	–	–	–	–	–	–	Scintigraphy	Very sensitive and quite specific marker for bone infection
Lee [35]	–	–	–	–	–	–	–	SPECT	Good method to detect polmonary tubercolosis
Pucar [54]	–	–	–	–	–	–	–	Scintigraphy	Good sensitivity but lack of specificity
Satpati [18]	2 h	5 × 10^7^—*S. aureus*	Right thigh	Muscle	3/6.6	1.5/2.2	Turpentine oil	–	Better (2) as bacteria-specific imaging agent
Oh [16]	4 h	2 × 10^8^—*S. aureus*	Right thigh	Muscle	3.7	–	–	–	Not enough data
Doroudi [17]	–	n. a.—*S. aureus*	Right thigh	Muscle	–	–	Carrageenan inflammation	Scintigraphy	High sensitivity, low specificity
Aungurarat [19]	1 h	n. a.—*S. aureus*, *P. aeruginosa*	Right thigh	Muscle	1.75	1.8	Turpentine oil, heat killed bacteria	–	Promising agent
Mirshojaei [22]	1 h	10^8^—*S. aureus*	Right thigh	Muscle	3.2	–	Sterile saline	Scintigraphy	Promising agent
Zhang [20]	4 h	4 × 10^10^—*Staphylobacterin*	Left thigh	Muscle	4.3	01:03	Turpentine oil	–	Sensitive radiopharmaceutical, simple preparation
Bhardwaj [14]	24 h	10^7^—*S. aureus*	Right thigh	Muscle	3.5	1.1	Turpentine oil	Scintigraphy	Stable, safe preparation, specific
Sarda [23]	–	10^7^—MS *S. aureus*	Joint	Knee	–	–	Sterile saline	Scintigraphy	Good sensitivity but lack of specificity for knee prosthesis
Peremans [26]	–	–	Joint	Knee	–	–	–	Scintigraphy	Useful for infected hip prosthesis diagnosis
Alexander [25]	–	–	–	–	–	–	–	Scintigraphy	Potential infection imaging agent
Wang [24]	–	–	–	–	–	–	–	Scintigraphy/CT	Better scintigraphy for pancreatites infection
Dahiya [21]	4 h	n. a.—*S. aureus*	Left thigh	Muscle	3.14	–	–	–	Potential infection imaging agent
Zhang [57]	4 h	10^10^—*S. aureus*	Left thigh	Muscle	1.78	04:28	^99m^Tc-ciprofloxacin	–	Better ciprofloxacin, potential imaging agent
Zhang [58]	4 h	10^10^—*S. aureus*	Left thigh	Muscle	4.47	4.28/1.78	^99m^Tc-ciprofloxacin/^99m^TcN-CPFXDTC	–	Potential infection imaging agent

We found 31 published studies in man (of which 18 were classified as “good”, 7 as “average” and 6 as “poor” on the basis of the reported diagnostic accuracy) and 14 studies in animals (of which 4 were classified as “good”, 7 as “average” and 3 as “poor” on the basis of the reported specificity to tested bacteria)

**Table 2:** Comparative analysis of paper published with other radiolabelled antibiotics in animals

Type of radiopharmaceutical	Radiopharmaceutical	First author (ref)	Labelling method	Specific activity (MBq/mmol)	Stability		Animal model	Metabolic route
					Saline	Serum		
Fluoroquinolones	^99m^Tc-gatifloxacin	M. A. Motaleb [95]	Direct labelling—SnCl_2_·2H_2_O	5.45 × 10^4^	81.3 % at 8 h	77.4 % at 24 h	Rat	Liver (+), kidneys (++)
	^99m^Tc-pefloxacin	E. A. El-Ghany [61]	Direct labelling—SnCl_2_·2H_2_O	6.53 × 10^5^	96 % at 12 h	n. a.	Mouse	Liver (++)
	^99m^Tc-ofloxacin	M. Erfani [63]	Carbonil core	6.05 × 10^2^	90 % at 6 h	80 % at 6 h	Mouse	Liver (++), kidneys (+)
	^99m^Tc-ofloxacin	M. A. Motaleb [62]	Direct labelling—SnCl_2_·2H_2_O	7.02 × 10^4^	96 % at 2 h	n. a.	Rat	Kidneys (++)
	^99m^Tc-lomefloxacin	M. A. Motaleb [62]	Direct labelling—SnCl_2_·2H_2_O	5.34 × 10^4^	80 % at 2 h	n. a.	Rat	Kidneys (++)
	^99m^Tc-enrofloxacin	R. H. Siaens [64]	Direct labelling-stannous tartrate	5.76 × 10^4^	n. a.	72 % at 24 h	Rat	Kidneys (++)
	^99m^Tc-enrofloxacin	S. Shahzad [65]	Direct labelling—SnCl_2_·2H_2_O	6.5 × 10^4^	98 % at 5 h	98 % at 5 h	Rabbit	Liver (+), kidneys (++)
	^99m^Tc-norfloxacin	I. T. Ibrahim [66]	Direct labelling—SnCl_2_·2H_2_O	2.53 × 10^4^	78.6 % at 6 h	84 % at 24 h	Rat	Liver (+), kidneys (++)
	^99m^Tc-norfloxacin	S. I. Sazonova [67]	Direct labelling—SnCl_2_·2H_2_O	n. a.	n. a.	91 % at 8 h	Rat	Liver (+), kidneys (++)
	^99m^TcN-norfloxacin dithiocarbamate	S. Zhang [68]	SnCl_2_·2H_2_O, succinic dihydrazide, propylenediamine tetraacetic acid	n. a.	96 % at 6 h	95 % at 6 h	Mouse	Liver (++), kidneys (+)
	^99m^Tc-sparafloxacin	M. A. Motaleb [9]	Direct labelling—SnCl_2_·2H_2_O	6.33 × 10^4^	n. a.	75 % at 24 h	Rat	Liver and kidneys
	^99m^Tc-rufloxacin	M. A. Motaleb [71]	Direct labelling—SnCl_2_·2H_2_O	2.52 × 10^3^	93.4 % at 8 h	82 % at 24 h	Mouse	Liver (++), kidneys (+)
	^18^F-fleroxacin	A. J. Fischman [72]	Potassius fluoride	n. a.	n. a.	n. a.	Mouse, rat, rabbit	Intestine (++), liver (+), kidneys (+)
	^99m^Tc-sitafloxacin	S. S. Qaiser [74]	Direct labelling—SnCl_2_·2H_2_O	2.17 × 10^4^	87.2 % at 4 h	n. a.	Rat	Liver (+), kidneys (++)
	^99m^Tc-levofloxacin	S. Shahzad [70]	Direct labelling—SnCl_2_·2H_2_O	1.35 × 10^5^	98 % at 6 h	98 % at 6 h	Rabbit	Liver (+), kidneys (++)
	^99m^TcN-sitafloxacin dithiocarbamate	S. S. Qaiser [75]	Direct labelling—SnCl_2_·2H_2_O	7.32 × 10^3^	91 % at 4 h	90 % at 4 h	Rat	Liver (+), kidneys (++)
	^99m^Tc-sarafloxacin	M. E. Moustapha [79]	Direct labelling—SnCl_2_·2H_2_O	1.47 × 10^5^	65 % at 8 h	85 % at 24 h	Mouse	Liver and kidneys
	^99m^Tc-moxifloxacin	S. Chattopadhyay [76]	Direct labelling—SnCl_2_·2H_2_O	1.05 × 10^4^	84 % at 3 h	n. a.	Rat/rabbit	Liver (+), kidneys(++)
	^99m^Tc-gemifloxacin	S. Shahzad [78]	SnCl2·2H2O-D-penicillamine	1.46 × 10^5^	98 % at 6 h	98 % at 6 h	Rabbit	Liver (+), kidneys(++)
	^99m^Tc-gemifloxacin	S. S. Qaiser [77]	Direct labelling—SnCl_2_·2H_2_O	2.15 × 10^4^	91 % at 4 h	94 % at 2 h	Rat	Liver and kidneys
Cephalosporins	^99m^Tc-cefazolin	M. El-Tawoosy [80]	Direct labelling—SnCl_2_·2H_2_O	1.63 × 10^5^	n. a.	n. a.	Mouse	Liver (++), kidneys (+)
	^99m^Tc-cefuroxime acetil	F. Yurt Lambrecht [81]	Direct labelling—SnCl_2_·2H_2_O	3.81 × 10^4^	n. a.	n. a.	Rat	Liver and kidneys
	^99m^Tc-cefuroxime	S. Chattopadhyay [82]	Direct labelling-stannous tartrate	8 × 10^4^	95 % at 5 h	n. a.	Rat	Liver and kidneys
	^99m^Tc-ceftizoxime	V. Gomes Barreto [84]	Na-dithionite	4.38 × 10^4^	n. a.	n. a.	Rat	Kidneys (++)
	^99m^Tc-ceftizoxime	P. H. N. Costa [85]	Na-dithionite	n. a.	n. a.	n. a.	Rat	n. a.
	^99m^Tc-ceftizoxime	L. E. M. Teixeira [86]	Na-dithionite	5.3 × 10^4^	n. a.	n. a.	Rat	n. a.
	^99m^Tc-cefotaxime	S. F. Mirshojaei [87]	Na-dithionite	9.4 × 10^4^	92 % at 12 h	85 % at 24 h	Mouse	Liver (+), kidneys (++)
	^99m^Tc-cefotaxime sodium	D. Ilem-Ozdemir [88]	Stannous tartrate/Stannous chloride	3.4 × 10^3^	92 % at 24 h	85 % at 24 h	Rat	Liver (+), kidneys (++)
	^99m^Tc-ceftriaxone	M. Mostafa [89]	Direct labelling—SnCl_2_·2H_2_O	1.90 × 10^2^	n. a.	n. a.	Mouse	Kidneys (++)
	^99m^Tc-ceftriaxone	A. Kaul [90]	Stannous tartrate and gentisic acid	8.95 × 10^4^	95 % at 24 h	95 % at 24 h	Rabbit	Liver (++), kidneys (+)
	^99m^Tc-ceftriaxone	A. Fazli [91]	Direct labelling—SnCl_2_·2H_2_O	n. a.	80 % at 24 h	71.2 % at 24 h	Mouse	Liver (+), kidneys (++)
	^99m^Tc-ceftriaxone	M. Sohaib [92]	Direct labelling—SnCl_2_·2H_2_O	6.5 × 10^3^	92.8 at 24 h	90.5 at 24 h	Rat	Liver (+), kidneys (++)
	^99m^Tc-ceftazimide	S. F. Mirshojaei [93]	Na-dithionite	1.17 × 10^5^	n. a.	85 % at 24 h	Mouse	Liver (+), kidneys (++)
	^99m^Tc-cefoperazone	M. A. Motaleb [94]	Direct labelling—SnCl_2_·2H_2_O	8.51 × 10^4^	98 % at 6 h	n. a.	Rat	Kidneys (++)
	^99m^Tc-cefepime	M. A. Motaleb [95]	Direct labelling—SnCl_2_·2H_2_O	3.92 × 10^4^	98 % at 8 h	86.8 % at 24 h	Rat	Liver (+), kidneys (++)
Inhibitors of nucleic acid synthesis	^99m^Tc-rifampicin	S. S. Qaiser [98]	Direct labelling—SnCl_2_·2H_2_O	5.48 × 10^4^	90 % at 2 h	n. a.	Rat	Liver and kidneys
	^99m^Tc-nitrofurantoin	S. S. Qaiser [100]	Direct labelling—SnCl_2_·2H_2_O	9.01 × 10^3^	90 % at 2 h	87.5 % at 2 h	Rat	Liver and kidneys
Inhibitors of bacterial cell wall synthesis	^99m^Tc-amoxicillin	S. K. Shahzadi [103]	SnCl_2_·2H_2_O-Sodium/Potassium pyrophosphate	7.25 × 10^4^	92 % at 6 h	92 % at 6 h	Rabbit	Liver (+), kidneys (++)
	^99m^Tc-alafosfalin	C. Tsopelas [105]	Direct labelling—SnCl_2_·2H_2_O	4.01 × 10^3^	n. a.	n. a.	Rat	Kidneys (++)
	^99m^Tc-vancomycin	S. Roohi [108]	Direct labelling—SnCl_2_·2H_2_O	1.35 × 10^5^	95 % at 6 h	n. a.	Rat	Liver (+), kidneys (++)
	[^18^F]fluoroacetamido-D-glucopyranose	M. E. Martìnez [106]	Microwave irradiation	1.80 × 10^4^	n. a.	n. a.	Rat	Liver and kidneys
Inhibitors of protein synthesis	^99m^Tc-kanamycin	S. Roohi [110]	Direct labelling—SnCl_2_·2H_2_O	3.62 × 10^4^	98 % at 6 h	96.4 % at 24 h	Rat	Liver (++), kidneys (+)
	^99m^Tc-doxycycline hyclate (DOX)	D. Ilem-Ozdemir [111]	Stannous tartrate-ascorbic acid	3.70 × 10^5^	90 % at 6 h	94 % at 24 h	Rat	Kidneys (++)
	^99m^Tc-erythromycin	I. Y. Abdel-Ghaney [112]	Direct labelling—SnCl_2_·2H_2_O	1.94 × 10^5^	97 % at 2 h	87 % at 24 h	Mouse	Liver (+), kidneys (++)
	^99m^Tc-clarithromycin	E. H. Borai [116]	Direct labelling—SnCl_2_·2H_2_O	1.5 × 10^5^	90 % at 2 h	90 % at 24 h	Mouse	Liver (+), kidneys (++)
	^99m^Tc-vibramycin	S. Hina [113]	Direct labelling—SnCl_2_·2H_2_O	7.8 × 10^5^	95 % at 12 h	98 % at 24 h	Rat	Liver (++), kidneys (+)
	^99m^Tc-azithromycin	M. H. Sanad [115]	Direct labelling—SnCl_2_·2H_2_O	1.44 × 10^5^	97.5 % at 2 h	85.5 % at 24 h	Mouse	Liver (+), kidneys (++)
	^99m^Tc-clindamycin	S. Hina [118]	Direct labelling—SnCl_2_·2H_2_O	1.56 × 10^6^	95 % at 5 h	92 % at 24 h	Rat	Liver (+), kidneys (++)
Others	6-[^18^F]-fluoromaltose	G. Gowrishankar [122]	Nucleophilic displacement	n. a.	n. a.	n. a.	Mouse	Liver and kidneys
	^68^Ga-triacetylfusarinine C (TAFC)	M. Petrik [124]	Direct labelling-sodium acetate	92 × 10^6^	n. a.	80 % at 2 h	Rat	Lungs
	^68^Ga-ferrioxamine E (FOXE)	M. Petrik [124]	Direct labelling-sodium acetate	3.4 × 10^6^	n. a.	90 % at 2 h	Rat	Lungs
	^99m^Tc-mebendazole	T. Inceboz [120]	Direct labelling—SnCl_2_·2H_2_O	2.3–4.6 × 10^−2^	n. a.	n. a.	Rat	Liver (++), kidneys (+)
	^99m^Tc-HQMADA	M. A. Motaleb [125]	Direct labelling—SnCl_2_·2H_2_O	765.8 (MBq/mg)	89.7 % at 8 h	83.4 % at 24 h	Mouse	Liver (++), kidneys (+)

Type of radiopharmaceutical	Max* T*/NT ratio							Final comment by the authors
	Time	Bacteria (CFU—strain)	Infection site	BKG	*T*/NT	Control experiment	*T*/NT (control)	
Fluoroquinolones	3 h	10^8^—*E. coli*	Left thigh	Muscle	4.5	Turpentine oil/heat killed bacteria	4.1/4.4	Low specificity
	24 h	n. a.—*E. coli*	Right thigh	Muscle	5.6	Turpentine oil	0.75	Good specificity
	2 h	10^8^—*S. aureus*	Right thigh	Muscle	2.02	n. a.	n. a.	Suitable radiopharmaceutical
	n. a.	10^5^–10^6^—*S. aureus*	Left thigh	Muscle	4.3	n. a.	n. a.	Specificity to be improved
	n. a.	10^5^–10^6^—*S. aureus*	Left thigh	Muscle	6.5	n. a.	n. a.	Good specificity
	22 h	5 × 10^8^—*S. aureus*	Left thigh	Muscle	4.8	Turpentine oil/heat killed bacteria	3.85/3.8	Non specific
	1 h	3 × 10^8^—*S. typhi*	Thigh	Muscle	4.8	Normal	n. a.	Suitable radiopharmaceutical
	2 h	10^7^–10^8^—*S. aureus*	Left thigh	Muscle	6.9	Turpentine oil/heat killed bacteria	4.5/6	Non specific
	18 h	10^9^—*S. aureus*	Left thigh	Muscle	2.87	Turpentine oil	1	Promising radiotracer
	4 h	10^8^—*S. aureus*	Left thigh	Muscle	3.46	Turpentine oil	1.23	Promising radiotracer
	30 min	10^5^–10^6^—*S. aureus*	Left thigh	Muscle	5.9	None	n. a.	Specificity to be improved
	3 h	10^5^–10^6^—*E. coli*	Left thigh	Muscle	8.5	Turpentine oil/heat killed bacteria	3/4.5	Specific
	n. a.	10^9^—*E. coli*	Right thigh	Muscle	n. a.	n. a.	n. a.	Promising radiotracer for PET imaging
	2 h	2 × 10^8^—*S. aureus*	Right thigh	Muscle	23.13	Turpentine oil	1.13	Specific at early stage
	1 h	3 × 10^8^—*E.coli*, *P. aeruginosa*	Thigh	Muscle	1.3/8.09	Normal	n. a.	Promising for lung, sinus bone infections
	90 min	10^5^—*S. aureus*	Left thigh	Muscle	7.6	Heat killed bacteria	1	Recommended imaging agent
	2 h	n. a.—*S. aureus*	Left thigh	Muscle	4.2	Turpentine oil/heat killed bacteria	3.4/3.3	Non specific
	1 h	10^6^–10^8^—*E. coli*	Left thigh	Muscle	1.62	n. a.	n. a.	Specificity to be improved
	4 h	3 × 10^8^—*S. typhi*, *P. aeruginosa*, *K. pneumoniae*	Thigh	Muscle	8/8.8/16	Normal	n. a.	Specific for respiratory tract infections
	90 min	n. a.—*S. pneumoniae*	Left thigh	Muscle	4.88	Heat killed bacteria	1.4	Specific for S. Pneumoniae
Cephalosporins	30 min	10^7^–10^8^—*S. aureus*	Left thigh	Muscle	8.57	Turpentine oil	1.4	Specific at early stage
	4 h	2 × 10^8^—*S. aureus*	Right thigh	Muscle	2.5	Turpentine oil	1.2	Specific
	3 h	10^8^—*E. coli*	Left thigh	Muscle	1.8	Normal	n. a.	Potential imaging agent
	6 h	2 × 10^8^—*E. coli*	Left thigh	Muscle	3.24	Zymosan	1.65	Specific
	n. a.	n. a.	n. a.	n. a.	n. a.	Bone wax	n. a.	Good for deep sternal infection
	n. a.	10^9^—*S. aureus*	n. a.	n. a.	n. a.	Sterile implant	n. a.	Specificity to be improved
	1 h	10^8^—*S. aureus*	Right thigh	Muscle	2.89	Normal	n. a.	Potential imaging agent
	1 h	4 × 10^10^—*E. coli*	Right thigh	Muscle	3.77	Turpentine oil	3.30	Non specific
	4 h	n.a.—*E. coli*	Left thigh	Muscle	5.6	Turpentine oil/heat killed bacteria	1.49	Good specificity
	24 h	10^7^—*S. aureus*	Left forearm	Right forearm	4.5	Turpentine oil	1.4	Specific
	3 h	10^8^—*S. aureus*	Left thigh	Muscle	3.39	Turpentine oil/heat killed bacteria	3.12/2.48	Potential imaging Agent
	4 h	10^8^—*E. coli*, *S. aureus*	Left thigh	Muscle	12.6/2.36	Turpentine oil	1.4	Specific
	1 h	10^8^—*S. aureus*	Left thigh	Muscle	1.4	n. a.	n. a.	Suitable radiopharmaceutical
	45 min	10^5^–10^6^—*S. aureus*	Left thigh	Muscle	4.66	n. a.	n. a.	Good specificity
	3 h	10^8^–*E. coli*	Left thigh	Muscle	8.4	Turpentine oil/heat killed bacteria	3.31/4.13	Specific
Inhibitors of nucleic acid synthesis	90 min	2 × 10^8^—methicillin-res *S. aureus*	Thigh	Muscle	7.34	Turpentine oil	1.20	Specific for MRSA
	90 min	10^8^—*E. coli*	Right thigh	Muscle	4.83	Turpentine oil/normal	1	Promising radiotracer
Inhibitors of bacterial cell wall synthesis	2 h	3 × 10^8^—*S. pneumoniae*	Thigh	Muscle	4.6	n. a.	–	Suitable radiopharmaceutical
	4 h	10^8^—*S. aureus*	Right thigh	Muscle	4.32	^99m^Tc-DTPA/^99m^Tc-WBC	1.93/20.09	Good for imaging osteomyelitis
	1 h	10^8^—*S. aureus*	Right thigh	Muscle	5.1	Turpentine oil	1.2	Enough specific
	n. a.	10^7^—*E. coli*	Right thigh	Muscle	n. a.	Turpentine oil	–	Specific
Inhibitors of protein synthesis	30 min	2 × 10^8^—*S. aureus*	Right thigh	Muscle	2.5	n. a.	–	Able to localize bacterial infection
	5 h	4 × 10^10^—*E. coli*	Right thigh	Muscle	2.24	n. a.	n. a.	Non specific
	30 min	10^5^–10^6^—*S. aureus*	Left thigh	Muscle	5	Turpentine oil	4.8	Non specific
	2 h	10^8^—*S. aureus*	Left thigh	Muscle	7.33	Turpentine oil/heat killed bacteria	3.1/3.26	Specific
	1 h	2 × 10^8^—*S. aureus*	Left thigh	Muscle	2.64	Turpentine oil/heat killed bacteria	1.80/2.15	Good bacterial imaging agent
	2 h	n. a.—*S. aureus*	Left thigh	Muscle	6.2	Turpentine oil/heat killed bacteria	2.60/3.13	Specific
	1 h	2 × 10^8^—*S. aureus*	Left thigh	Muscle	3.1	Turpentine oil/heat killed bacteria	2.47/1.6	Potential imaging agent
Others	n. a.	5 × 10^7^—*E. coli*	Right thigh	Muscle	n. a.	Turpentine oil/heat killed bacteria	–	Specific
	n. a.	10^9^—*A. fumigatus*	Left calf	Muscle	n. a.	Turpentine oil	n. a.	Specific for A. Fumigatus
	n. a.	10^9^—*A. fumigatus*	Left calf	Muscle	n. a.	Turpentine oil	n. a.	Specific for A. Fumigatus
	n. a.	750–1000 larvae *T. spiralis*	Diaphragm	Muscle	n. a.	n. a.	n. a.	Specific for T. Spiralis
	2 h	n. a.—*E. coli*	Left thigh	Muscle	5.52	Turpentine oil/heat killed bacteria	2.5/2.2	Potential substitute of ciprofloxacin

We found 53 published studies in animals and none in man (of which 18 were classified as “good”, 23 as “average” and 12 as “poor” on the basis of the reported specificity to tested bacteria)

Ciprofloxacin studies were selected and used as comparison to other antibiotics because ciprofloxacin was the first antibiotic tested in humans. As shown in Table 1, many groups worldwide obtained conflicting results in terms of sensibility and specificity. Overall, in animal models ciprofloxacin showed good sensibility but a lack of specificity, probably because of labelling issues and poor stability. In clinical studies data are more complicated to analyse because different authors used different scoring systems that may result subjective to interpretation [29], as it can be seen in Table 1. A multicentre study with homogeneous criteria of image acquisition and interpretation is still missing.

Table 2 shows the results of our analysis of all other radiolabelled antibiotics. None was studied in man. It is possible to note that very often different studies are performed by the same group of authors. Overall studies are published in journals with low impact factor and relevance. There is low reproducibility and reliability because most antibiotics were not tested by more than one team. Sometimes reports are incomplete with no in vitro and/or in vivo data and it was not possible to analyse all experimental results. It is anyhow remarkable to observe the variability of labelling procedure, and the high variability of specific activity of the radiopharmaceutical. Often stability in serum or saline are not performed or not for enough time. Animal models are variable and the type of bacteria used and CFU injected is extremely variable. In particular the number of bacteria used may be relevant because higher numbers can give a higher signal by binding more molecules of radiopharmaceutical [7].

Mainly the infection was induced using *S. aureus* or *E. coli*, except when the antibiotic was specific for a certain bacterium such as *A. fumigatus* or *T. spiralis*. The metabolism of most radiopharmaceuticals is renal and rarely hepatic. The specificity is related to in vivo calculated target to background ratio (*T*/NT) using turpentine oil and/or heat killed bacteria as control. Very rarely we found in vitro data on binding to bacteria or ex vivo autoradiography to demonstrate the specificity of binding to bacteria. Most* T*/NT ratios were below 4 (poor radiopharmaceuticals), a few were between 4 and 8 (promising radiopharmaceuticals), and only ^99m^Tc-cefazolin, ^99m^Tc-cefepime, ^99m^Tc-clarithromycin, ^99m^Tc-rufloxacin, ^99m^Tc-ceftriaxone, ^99m^Tc-levofloxacin, ^99m^Tc-gemifloxacin and ^99m^Tc-sitafloxacin showed a* T*/NT ratio higher than 8 (good radiopharmaceutical). This indicates that most radiolabelled antibiotics are not candidate for human studies.

## Conclusion

From the present systematic review it can deduced how difficult it is to find a specific imaging agent to detect bacterial infection and to monitor the effectiveness of antimicrobial therapy. None of the mentioned radiolabelled antibiotics is commercially available because of its minimal or very low specific activity or low specificity for infections versus sterile inflammation or, most frequently for selective specificity to one kind of bacteria only. Despite a large number of original papers have been published, it is difficult to make a head-to-head comparison amongst them. Animal models are often different (mice, rats or rabbits), injected activities and image acquisition times are different, and, most importantly, the number of bacteria used for inducing the infection ranges from 10^5^ to 10^10^, being the main limiting factor for a comparison of sensitivity.

Another important problem of antibiotics is the risk of resistance mechanism because bacteria can change very quickly and drug-resistant strains are often the cause of recurring infections. Resistance can also be due from a non specific removal mechanism of antibiotics or sometimes from an enforced efflux by pumps. Furthermore, bacteria do not have a high affinity for antibiotics, nor the binding between the antibiotic and bacteria is specific like the ligand-receptor interaction in mammalian cells. For these reasons the gold standard for bacterial infection imaging has not yet been found. Hopefully in future we will have many radiopharmaceuticals available, tailored for specific pathogens, and clinical conditions thus having the maximum specificity.

It is important also to stress that animal experiments should always be performed before human studies, with several different strains and number of bacteria in order to provide useful information for planning and interpreting human studies.
